# Autonomous digitizer calibration of a Monte Carlo detector model through evolutionary simulation

**DOI:** 10.1038/s41598-022-24022-x

**Published:** 2022-11-14

**Authors:** Matthew Herald, Andrei Nicuşan, Tzany Kokalova Wheldon, Jonathan Seville, Christopher Windows-Yule

**Affiliations:** 1 School of Chemical Engineering, University of Birmingham, Birmingham, UK; 2 School of Physics and Astronomy, University of Birmingham, Birmingham, UK; 3 Positron Imaging Centre, University of Birmingham, Birmingham, UK

**Keywords:** Electronics, photonics and device physics, Nuclear physics, Techniques and instrumentation, Software

## Abstract

Simulating the response of a radiation detector is a modelling challenge due to the stochastic nature of radiation, often complex geometries, and multi-stage signal processing. While sophisticated tools for Monte Carlo simulation have been developed for radiation transport, emulating signal processing and data loss must be accomplished using a simplified model of the electronics called the digitizer. Due to a large number of free parameters, calibrating a digitizer quickly becomes an optimisation problem. To address this, we propose a novel technique by which evolutionary algorithms calibrate a digitizer autonomously. We demonstrate this by calibrating six free parameters in a digitizer model for the ADAC Forte. The accuracy of solutions is quantified via a cost function measuring the absolute percent difference between simulated and experimental coincidence count rates across a robust characterisation data set, including three detector configurations and a range of source activities. Ultimately, this calibration produces a count rate response with 5.8% mean difference to the experiment, improving from 18.3% difference when manually calibrated. Using evolutionary algorithms for model calibration is a notable advancement because this method is novel, autonomous, fault-tolerant, and achieved through a direct comparison of simulation to reality. The software used in this work has been made freely available through a GitHub repository.

## Introduction

Simulating the response of detectors to radiation is an important aspect in a variety of physics and medical fields because this allows users to test imaging algorithms, optimise experiments, and design new detectors^1–3^. This is typically achieved by using Monte Carlo radiation transport codes to simulate the interactions of a radiation field with a geometric model of the detector and then applying a pulse-processing chain to the recorded events to emulate the detector’s response^4^. Software such as the Geant4 Application for Tomographic Emission (GATE) has been developed specifically for the purpose of running Monte Carlo simulations and emulating detector responses^5,6^. In GATE, the ‘digitizer’ determines how the timing, energy, and position of interactions with the detector geometry are recorded, how events are grouped and implements the pulse-processing logic of the system^7^. However, digitizer models must be precisely tuned to replicate the behaviour of a real detector.

Several detectors have been modelled using GATE and validated against experimental measurements such as the ADAC Forte, Siemens Inveon, and Phillips Vereos Positron Emission Tomography (PET) scanners^8–10^. For PET systems, performance characterisation experiments are described by the National Electronics Manufacturers Associated (NEMA) which test the spatial resolution, sensitivity, and count-rate response^11^. The GATE model’s digitizer is then calibrated to achieve the closest agreement with these experiments. GATE models which do not have well-calibrated digitizers may produce an unrealistic simulated detector response.

### State-of-the-art

Current methods of calibrating GATE models, as demonstrated in other work, are achieved by using known properties of the detector or by fitting models to count-rate experiments^7,10,12,13^. Many steps in the digitizer model correspond directly to measurable properties of the detector, such as the energy resolution, dead-time, or time resolution^10^. Values for these properties are often provided by the manufacturer and this can serve as a reliable starting point, but manual tuning is still needed to match the simulated and experimental response of the detector due to variation between each detector^8^. Conversely, with a model-fitting approach, manual tuning can be avoided, but other challenges arise. For example, when fitting a dead-time model to the count rates or fitting a Gaussian function to the 511 keV photo-peak to determine the energy resolution, this relies on having both the singles and coincidence count rates, which may not both be available, and also involves fitting simplified models to the detector response, which may not capture the complexity of a real system^14^. In summary, manual tuning of GATE models can produce a good agreement between simulation and experiment, but at the expense of time, resources, and objectivity, whereas fitting simplified models to determine the digitizer parameter values is a quicker, more objective, method but the information is not always available and can still produce inaccurate simulations.

### Proposed methodology

In this work, we propose a new procedure which leverages recent advances in metaheuristics to perform an efficient optimisation of parameter values in a detector digitizer model created using GATE v9.1. The goal of the optimisation is to produce a set of parameters which can replicate the count-rate response of the detector across varied source activities and detector separations.

**Figure 1:**
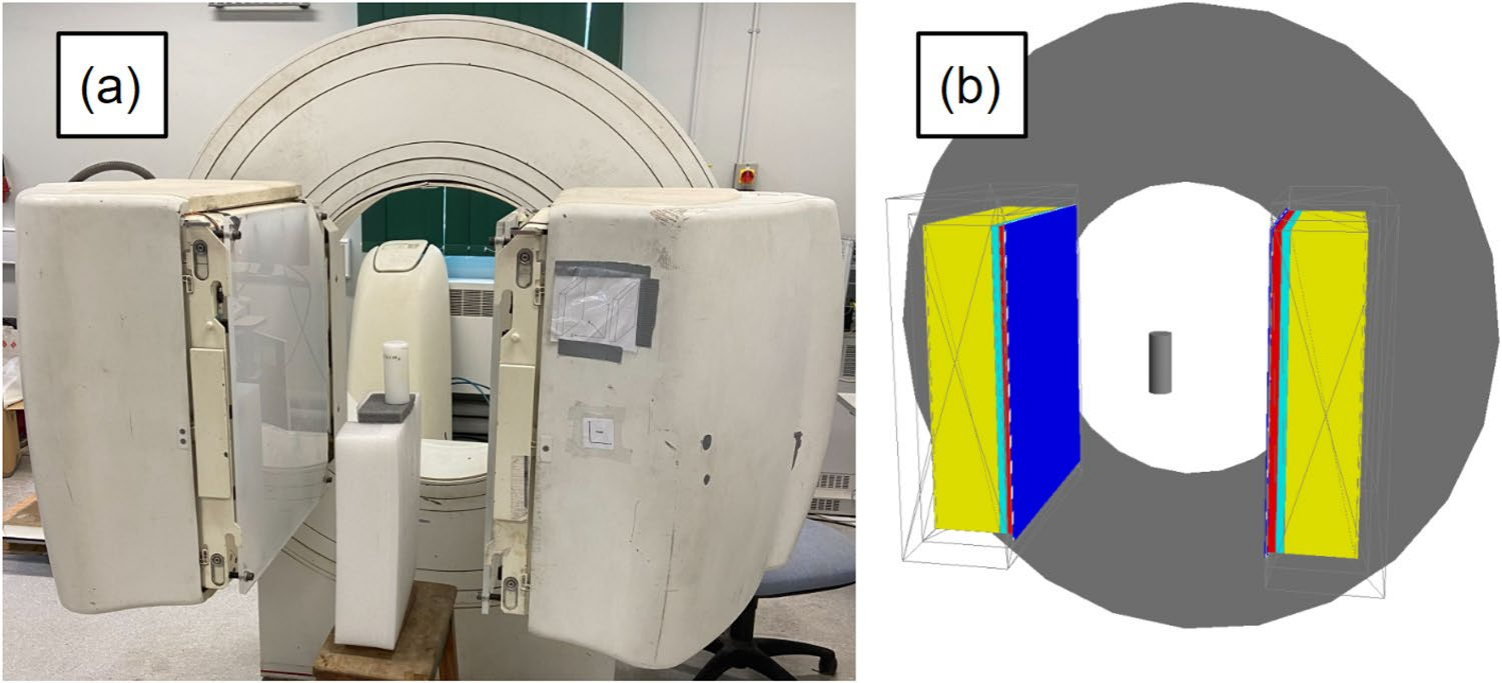
The ADAC Forte at the Positron Imaging Centre during the count-rate experiment (**a**) and the GATE model of the detector and replicated experiment (**b**).

To do this we use an evolutionary algorithm to modify the free parameters of the digitizer , resembling Darwinian evolution, and directly compare the simulated results of candidate solutions to the experimental data. The evolutionary algorithm chosen for this approach is the Covariance Matrix Adaptation Evolutionary Strategy (CMA-ES), which is a stochastic optimiser for robust non-linear non-convex numerical optimisation^15,16^. Parameter combinations are generated following a multivariate normal distribution; in our case, finding the optimum digitizer parameters is equivalent to “evolving” the mean and covariance matrix of this distribution. A particular advantage of this setup is that the underlying optimisation function – i.e. the digitizer response – does not need to have a continuous response. The addition of stochastic “mutations” to the inputs tried, so as to mimic the injection of new genetic material in the biological population, allows CMA-ES to escape local, false minima, which gradient-based optimisers are prone to falling into^17^. We demonstrate this procedure by calibrating the GATE digitizer model of the ADAC Forte, a dual-headed positron camera operated in coincidence mode^8^. The Forte and its digital-twin GATE model are shown in Fig. 1. Six free parameters in the model are calibrated simultaneously by CMA-ES.

In order to interface with the existing CMA-ES optimiser and extend the types of problems it can be used with, we have developed a Python library called the Autonomous Calibration and Characterisation via Evolutionary Software (ACCES) v0.2.2. The purpose of ACCES is to use meta-programming in conjunction with an arbitrary Python script defining the simulation to populate the user-defined free parameters with candidate solutions generated by CMA-ES, then autonomously re-launch the simulation, analyse the results, and use CMA-ES to generate candidate solutions in a cycle until a termination criterion is met^16,18^. The absolute percent difference between the total, true, and scattered plus random coincidence count rates are optimised using a multi-objective cost function to combine their differences into a single value. This method offers improvements over previous calibration procedures since the optimal parameters are chosen by directly comparing the performance of the optimised digitizer to count-rate experiments and multiple experiments are optimised simultaneously.

## Methods

### Count rate experiment

Characterisation experiments are conducted that measure the coincidence count rates of the Forte as a function of source activity. The experimental coincidence count rates are chosen to be compared to the simulated count rates to assess the optimisation of the model since this is the observable output from the detector in experiments and simulations. Additionally, the coincidence count rates have a complex relationship to the digitizer parameters, source activity, and detector configuration making this an ideal metric for comparison. Three detector separations representing the closest, median, and furthest separations possible for the detector are tested. The initial source activities for each separation are selected to test both the high-activity range where the effect of detector dead-time induces count-losses and, as the source decays over several half-lives, testing the low-activity range where count-rates are linearly proportional to the source activity. The optimisation of the digitizer seeks to find a common set of parameter values to replicate the behaviour of the detector across all of these conditions. Since there are six free parameters and 45 individual data points for each parameter combination (three detector separations, five activities per separation, and three coincidence count rates per activity), the optimisation problem is considered to be well-constrained.

For these experiments, the source consists of a high-density polyethene (HDPE) cylindrical phantom filled with a solution of water and fluorine-18. The phantom measures 120 mm long and 50 mm in diameter. The inner cylinder in which the water and fluorine-18 solution is filled measures 100 mm long and 12 mm in diameter. The phantom is filled with an initial activity, then placed in the centre of the field-of-view of the Forte and imaged over several half-lives until the activity is below 1 MBq. The three head separations and initial activities for each experiment are found in Table 1.

**Table 1:** Head separations and initial activities for each calibration experiment.

Experiment	Head separation (mm)	Initial activity (MBq)
Experiment 1	800	75
Experiment 2	525	60
Experiment 3	250	40

**Figure 2:**
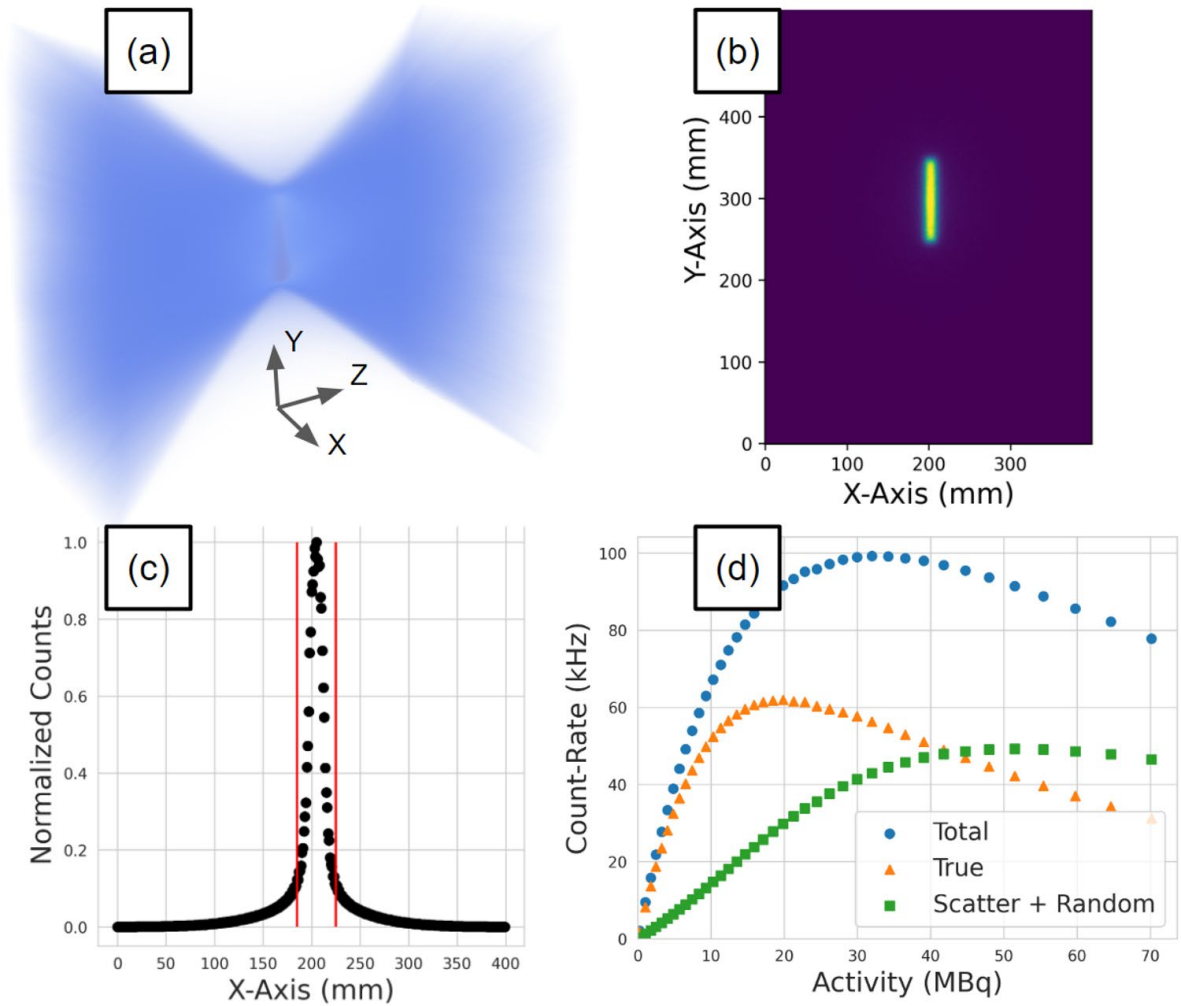
A demonstration of the protocol for extracting count rates from a sample of LoRs: (**a**) a sample of LoRs collected during the experiment is converted into voxels, (**b**) the slice containing the maximum number of LoRs is extracted, (**c**) the slice is collapsed into a line profile and the counts in the central 40 mm strip are summed and background counts subtracted to yield a total, true, and scattered + random count-rate (**d**). Steps a-c are repeated for multiple samples to generate the count-rate response as a function of activity.

**Figure 3:**
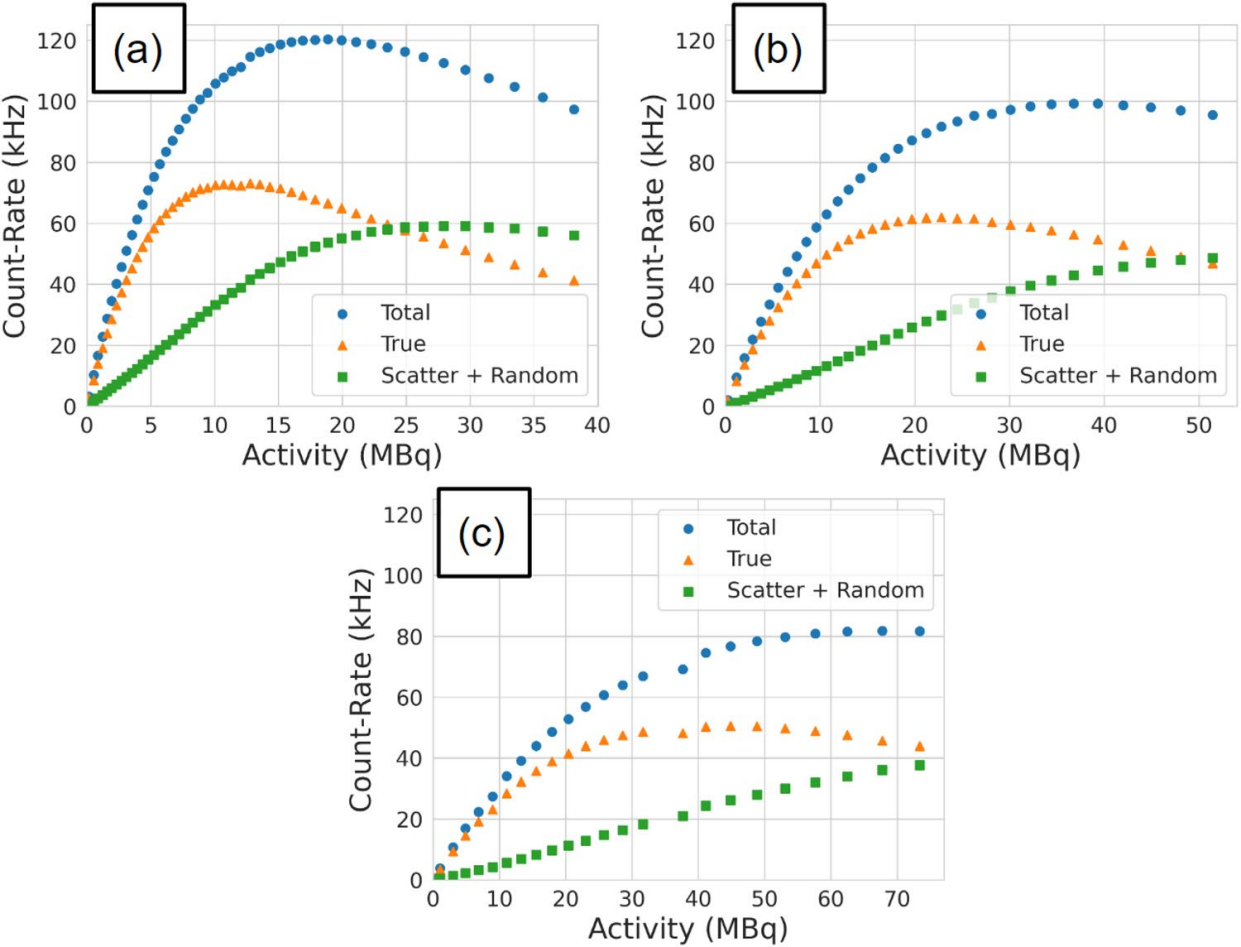
The total, true, and scatter + random coincidence count rates as a function of source activity for (**a**) 250 mm, (**b**) 525 mm, and (**c**) 800 mm head separation.

For each experiment, the total, true, and scattered plus random (corrupted) count rates are extracted as a function of the source activity. This is achieved by applying the NEMA protocol to projection images of the source^11^. A demonstration of the workflow for extracting count rates from the acquisition is shown in Fig. 2. First, samples of a minimum of 500,000 lines-of-response (LoRs) are used to create a three-dimensional voxelised representation of the FOV with a 1 mm voxel size. At this stage, the source activity is calculated using exponential decay equations. From the voxels, a two-dimensional slice is extracted which is both parallel with the detector face and contains the voxel with the maximum number of LoRs. The slice is then collapsed into a line profile of the pixel intensities. All points within ± 20 mm of the maximum pixel are summed. To subtract the background counts, the values at both ends of the ± 20 mm are averaged, multiplied by the size of the window, and subtracted from the counts under the peak leaving only the true counts. The total counts are the sum of all LoRs passing through the slice and the scattered plus random coincidence count rate is the total counts subtracted by the true counts. The extracted coincidence count rates are shown in Fig. 3.

### GATE model

GATE v9.1 is an extension of Geant4 v10.7.3 designed for the simulation of radiation detectors used in physics, medicine, and engineering applications^5,6^. GATE uses Geant4 to run Monte Carlo radiation transport simulations, generating a history of interactions of the detector with a radiation field, and then mimicking how the detector would respond^19^. Using GATE consists of 6 steps: defining the geometries (detector and experiment), adding radioactive sources, describing the detector pulse processing chain (digitizer), including physics processes, specifying data output format, and prescribing acquisition settings (run time and time slice)^5^.

In this work, we use a detector geometry and GATE model for the ADAC Forte previously developed by the authors. The ADAC Forte is a dual-headed positron camera used at the Positron Imaging Centre^8^. A full description of this model and its original calibration can be found in Herald et al.^8^. The experiment geometry is the same HDPE cylindrical phantom as described in “Count rate experiment” section. The radioactive source is a solution of water and fluorine-18 prescribed as emitting back-to-back 511 keV gamma rays. Since the mean positron range in water and HDPE it can be assumed that all positrons annihilate before leaving the phantom, thus making a back-to-back gamma source a reasonable approximation that decreases the time needed to run the simulations. The detector model’s digitizer structure follows the same as described in Herald et al., (2021). Six key parameters of the digitizer will be calibrated. These are the singles dead-time, coincidence dead-time, pileup, lower energy discriminator, upper energy discriminator, and the time resolutions as will be discussed is 2.2. Physics processes are imported through the GATE’s ‘emstandard’ physics list, which includes the Livermore model for photon interactions and is based on the Evaluated Photon Data Library, 1997 (EPDL1997)^20^. The output format is coincidence data saved as a text file. The acquisition was prescribed as a 10 second simulation with the time slice saving data every 10 ms of simulated time.

**Figure 4:**
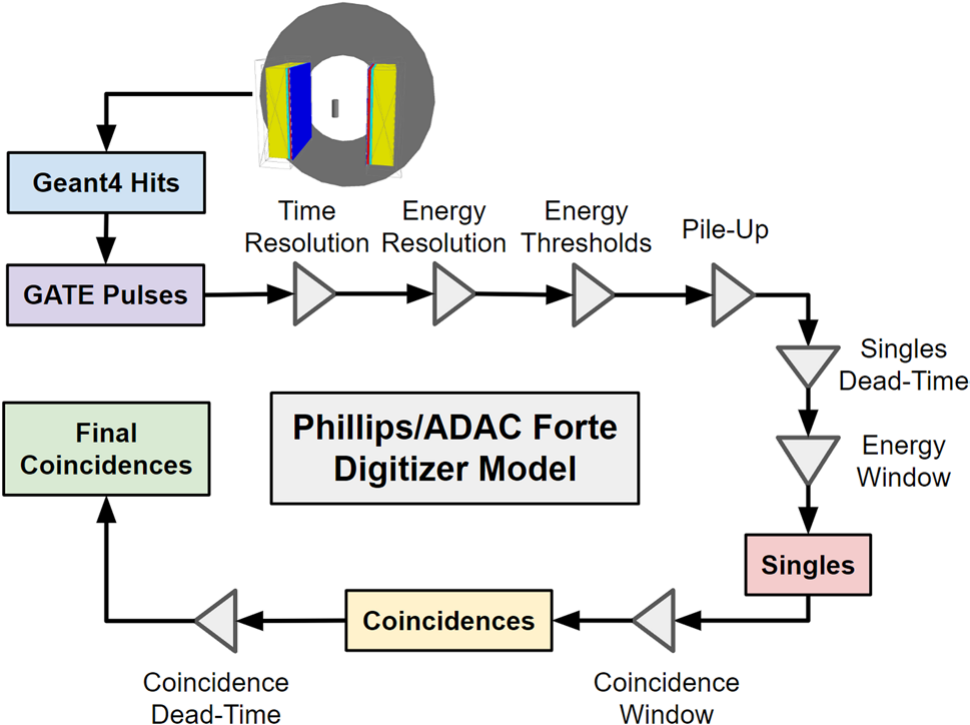
The digitizer model of the ADAC Forte.

Once the simulation begins the source activity determines the decay rate and individual decays are modelled on a Poisson distribution. Each event (two back-to-back 511 keV gamma rays) is initialised randomly within the source volume and prescribed a direction isotropically. As the gamma rays pass through the geometry, they have a stochastic chance of interacting with the materials following Beer-Lambert’s Law and using attenuation coefficients generated from material composition, density, and cross-sections from EPDL1997. Interactions which occur within the ‘Sensitive Detector’, in this case, the scintillation crystals, are termed ‘hits’. From the list of hits, which contains information about the type of interaction, time, position, and energy, the GATE digitizer converts hits into ‘pulses’. A pulse is the response of the detector element that is analogous to a signal which can be processed, eventually producing an output of what a real detector would record. The digitizer model for the ADAC Forte is shown in Fig. 4.

In this work, we demonstrate a novel application of evolutionary algorithms to calibrate the digitizer for a GATE model of the ADAC Forte, a dual-headed positron camera used at the Positron Imaging Centre^8^. The primary use of this GATE model is to emulate experiments using positron emission particle tracking, a radio-imaging technique, in order to estimate the spatial and temporal resolution of tracer trajectories and to optimise experiment design^21,22^. The detector consists of two wide-area sodium-iodide crystals measuring 590 mm $\times$ 470 mm and 16 mm thick^23^. The active area for recording coincidences measures 510 mm $\times$ 380 mm. Additionally, the two detector heads can be moved between 250 mm and 800 mm of separation which can accommodate a variety of experiments^24,25^. The Forte and the GATE model of the Forte are shown in Fig. 1.

The six digitizer parameters chosen to be optimised are the singles dead-time, coincidence dead-time, pileup, lower energy discriminator, upper energy discriminator, and the time resolutions which are explained below. These parameters were chosen because they have not been measured directly through a characterisation experiment meaning there is uncertainty in the optimal values. The singles dead-time is a paralysable dead-time which affects each pulse, rendering the detector unable to record another pulse until the dead-time has ended^14^. If another gamma ray enters the detector before the singles dead-time is completed, the dead-time is reset and the gamma-ray is not recorded. Paralysable dead-time results in count losses and at high source activities can cause the count rate to decrease. Coincidence dead-time is a separate, non-paralysable dead-time affecting the recording of a coincidence^26^. Unlike a paralysable model, a non-paralysable dead-time does not get reset with additional events. Pile-up time is the time between the detection of a single gamma-ray triggering the recording of the pulse and the time at which other events can ‘pile-up‘ onto the same pulse^14^. Pile-up has the effect of creating count-losses at high source activities. The lower and upper energy discriminators are the minimum and maximum energies of events which can trigger the singles dead-time^27^. The time resolution is the uncertainty in the timing of precision of the detector, defined by a Gaussian blurring with a full-width half-maximum^28^. If two gamma rays interact with the detector within the coincidence window of 15 ns, they are not guaranteed to be detected in coincidence due to the timing uncertainty. This has the effect of disregarding some real coincidences and accepting more random coincidences.

### ACCES

When trying to calibrate a simulation’s free parameters so that an experimental measurement can be replicated, it is often useful to test a range of conditions and assess how the tested parameter values replicate the measurement. In the simplest case with only one free parameter, the value that minimises the error to the measurement can be easily found and visualised by plotting parameter values and the error as a two-dimensional plot. This can also be extended to two free parameters by plotting the error as a third dimension on the plot. Beyond three dimensions, the number of parameter values needed to explore the solution space increases exponentially and the relationship between the parameters becomes non-intuitive. For these problems, an optimiser is needed to efficiently test a range of parameter values and converge to a set of optimal parameter values. However, in simulations and experiments, there often exist noisy measurements, thus a function defining the difference between experiment and simulation will be non-smooth and potentially have many false local minima. This means that gradient-based optimisers are ill-suited for calibrating simulations.

**Figure 5:**
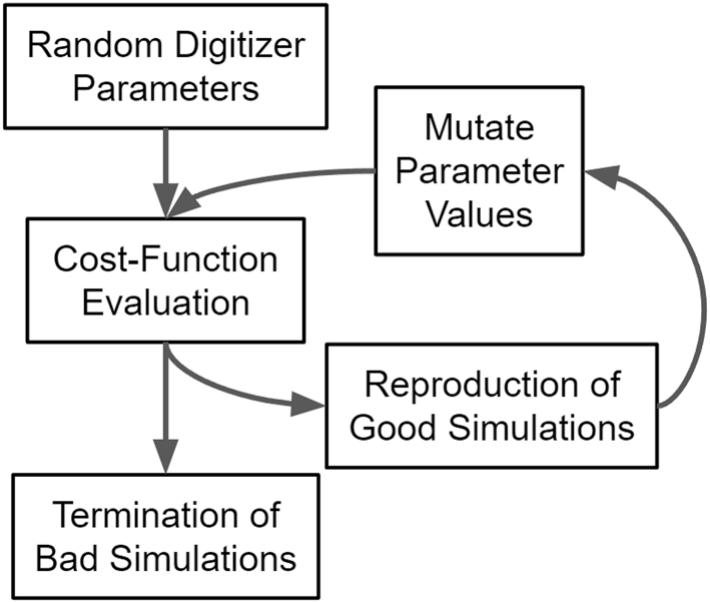
The flow diagram for ACCES is applied to optimising free parameters in a digitizer of a GATE detector model.

In these difficult optimisations, evolutionary algorithms excel^29^. Evolutionary algorithms are a type of bio-inspired computing which mimics natural selection. For example, in a population where individuals have a randomised set of genes and selective pressure is exerted, only the individuals which have genes that enable them to survive will reproduce. Due to this, the next generation of individuals will be more adapted to selective pressure. Similarly, when an evolutionary algorithm is applied to a model function with quantitative free parameters which can be tuned, the parameter values act as genes, a model with a specific set of parameters is an individual, and a group of individual simulations is a generation^29^. For each generation, a cost function determines an individual’s fitness and acts as a selective pressure. Using this method, parameter value combinations which result in a low cost function are prioritised until the solutions converge to a set of optimal values. A flow diagram of how an evolutionary algorithm can be applied to digitizer calibration is shown in Fig. 5.

While there exist several types of evolutionary algorithms the CMA-ES algorithm is used in this work since it performed well in a comparative review of optimisation algorithms and there is a well-documented Python implementation CMA v3.0.3^16,30^. To use CMA-ES, the ACCES Python library v0.2.2 is employed to interface with CMA-ES and edit an arbitrary script for updating free parameter values in the simulation^18^. Using code inspection and meta-programming, simulation scripts are parallelised by ACCES allowing them to be launched locally or on a high-performance computer. The difference between the simulated system and experimental reality can then be quantified by a cost function so that CMA-ES can determine the next generation of solutions. ACCES offers improvements over other interfaces to optimisers in that it is fault-tolerant and designed for high-performance computing.

ACCES needs only the bounds of the search parameters, and the number of individuals in a population, and stores the results after each generation, or ‘epoch’, so that the optimisation state can be restored at any point. The default implementation of CMA-ES requires the use of a single initial standard deviation for all parameters - i.e. assuming that all parameters have comparable value ranges and sensitivities. ACCES scales the parameter values by 40 % of each parameter’s allowed range, such that parameters of vastly different scales can be optimised together - e.g. singles dead times in the range [0, 2] and pile up between [0, 600]. As parameter combinations are drawn from normal distributions, an initial standard deviation of 40 % naturally covers the entire parameter range.

In order to allow the use of complex, potentially thread-unsafe simulations written in different programming languages, ACCES launches each simulation as a completely separate OS process, which is either scheduled by the kernel to be run locally on a shared-memory machine (e.g. a laptop) or using an external workload manager to launch jobs on multi-node clusters; in this study, ACCES automatically sets up and launches batch jobs for each parameter combination to be evaluated using GATE. To summarise, the two critical CMA-ES configuration parameters are automatically determined by the computing resources available and the possible parameter ranges, such that no manual adjustments of optimiser settings for a given problem is necessary.

### Digitizer calibration

We use ACCES in this work to optimise the six free parameters within the digitizer of the Forte GATE model described in “GATE model” section. The experiments described in “Count rate experiment” section are used to determine the fitness of parameter combinations. Specifically, a cost function is applied which measures the percent difference between the experimentally observed and simulated count rates for the total, true, and scatter plus random count-rates across all three head separations and activities. The sum for each of these percent differences is denoted as $\varepsilon _R$, $\varepsilon _T$, and $\varepsilon _{SR}$ respectively and computed using Eq. (1). Each type of count rate is treated as an objective to optimise and combined into a multi-objective optimisation by multiplying them together using Eq. (2). In this case, each type of count rate is treated as equally important; this could be changed by adding weights to each percent difference.

1 $$\begin{aligned}&\varepsilon _R = \sum 100\frac{|R_{exp}-R_{GATE}|}{R_{exp}} \end{aligned}$$

2 $$\begin{aligned}&\varepsilon = \varepsilon _R\varepsilon _T\varepsilon _{SR} \end{aligned}$$

**Figure 6:**
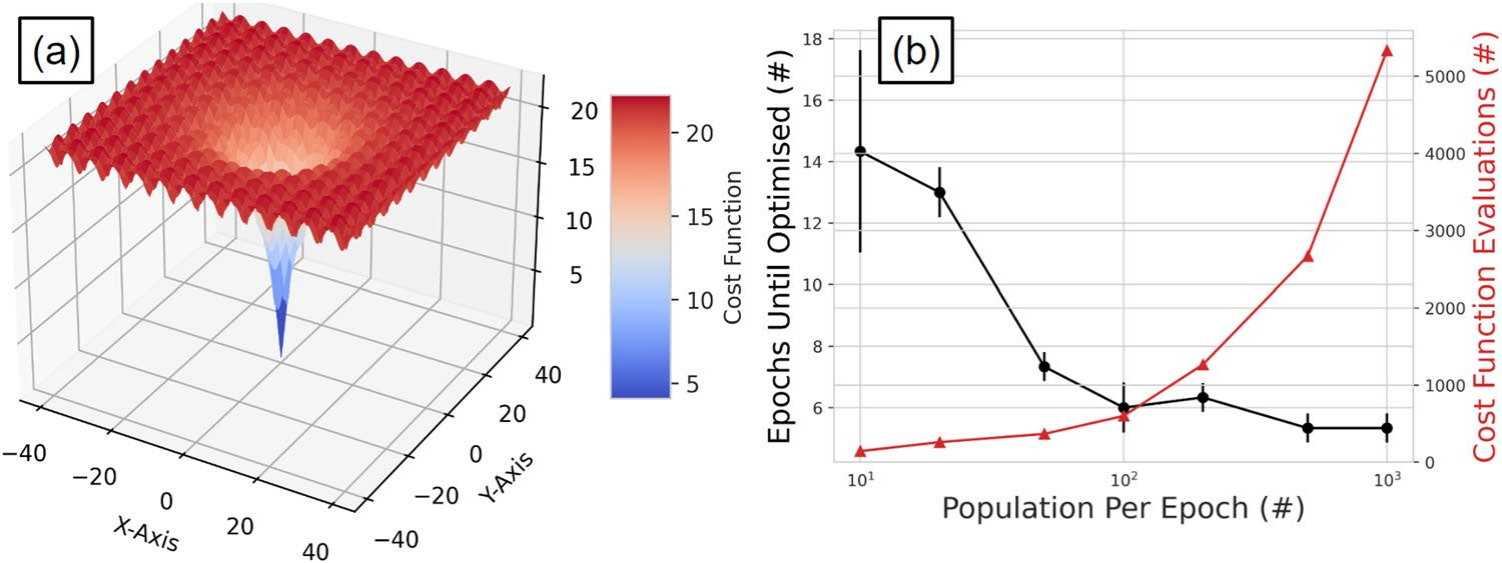
(**a**) The two-dimensional Ackley function with the with evaluation as the third dimension. (**b**) The behaviour of the number of epochs needed to find the global minimum as well the total number of cost functions evaluated as function of the number of solutions per epoch.

To run ACCES, three things must be prescribed: the number of simulations per epoch, the bounds of the parameter guesses, and the terminating criterion. The number of simulations per epoch should be large enough that sufficient learning can occur and the bounds of parameters must be set so as to keep guesses within a realistic range. We have tested ACCES using a simple analytical cost function, the Ackley function, which is widely used for testing optimization algorithms^31^. This function, described in Eq. (3), has many local minima and one global minimum. The number of epochs needed to find the global minimum as well as the total number of cost functions evaluated can be studied as a function of the number of solutions per epoch. The results from this study are shown in Fig. 6. We used a two-dimensional ($\hbox {d}=2$) Ackley function with the parameters $\hbox {a}=20$, $\hbox {b}=0.2$ and $\hbox {c}=2\pi$.

3 $$\begin{aligned} \varepsilon (x_i) = -a\exp {\left( -b\sqrt{\sum _{i=1}^{d}\frac{1}{d}x_{i}^2}\right) } - \exp {\sum _{i=1}^{d}\frac{1}{d}cos{(cx_{i})}} +a +\exp {(1)} \end{aligned}$$

The results from this show that larger numbers of solutions optimise the parameters in fewer epochs, but at a cost of increasing the number of cost function evaluations. When optimising the Monte Carlo model’s digitizer, in order to be computationally efficient, the lowest reasonable number of simulations per epoch should be run. The number of simulations is set to 150 so that a wide range of different parameter combinations can be tried and the simulations can all be launched in parallel while not affecting the shared usage of the BlueBEAR high-performance computing (HPC) system. In this work, each set of simulations with a common set of parameter solutions is run on a single Intel Icelake core of the BlueBEAR HPC with 8 GB of memory allocated. The maximum run-time is set to 4 hours and 30 minutes, which is approximately twice as long as the mean run-time expected. In the event that a set of simulations takes longer than 4 hours and 30 minutes, the job is terminated and the results are not in the solution space for the next generation of parameter solutions.

Additionally, the bounds of the parameter guesses are set to only explore solutions which make physical sense, excluding options like a negative dead-time or upper energy level being below the upper energy window. The bounds are also limited where needed such that the solution space is finite, yet spanning a range likely to contain the optimal value based on an estimate from a previously calibrated system^8^. A list of the bounds and the initial guesses are shown in Table 2.

**Table 2:** Digitizer parameter bounds and initial guesses.

Parameter	Lower bound	Upper bound	Initial guess
Singles dead-time (ns)	0	2	1
Coincidence dead-time (ns)	0	2	1
Pile-up (ns)	0	600	300
Lower energy discriminator (keV)	0	360	180
Upper energy discriminator (keV)	640	1200	920
Time resolution (ns)	10	20	15

**Figure 7:**
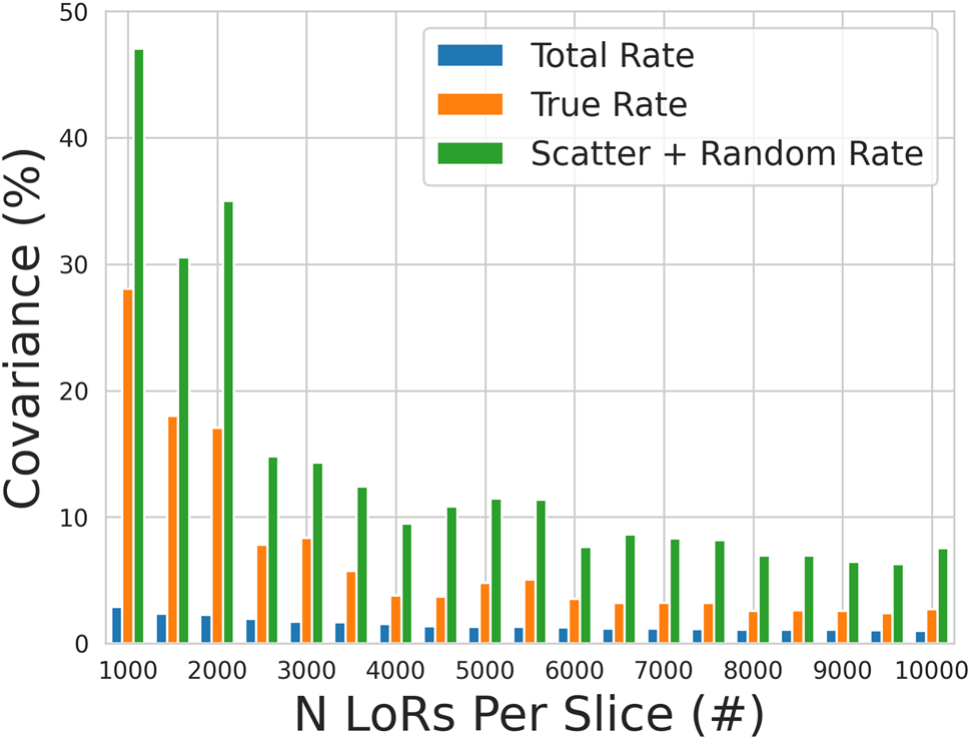
The covariance of the total, true, and scattered plus random count rates for the 800 mm and 2 MBq simulation as a function of the number of LoRs used to generate a projection image.

The termination criterion for the optimisation is the standard deviation for each parameter reaching 10% of the initial standard deviation. This range is chosen such that variation in the parameter values will not significantly affect the accuracy of the model. The initial standard deviation is equal to the range of the bounds at the beginning of the optimisation and the scaled standard deviation is defined as unity. Once the optimal values are identified, they are input to the digitizer model and a coincidence count-rate response is generated to compare with the experimental data. These simulations are run at 2 MBq intervals starting at 1 MBq and reaching into the upper activities for each experiment. A study of the accuracy of extracted count-rates for the simulation with the highest separation and lowest activity (800 mm and 2 MBq) at different numbers of LoRs used to produce projection images showed that at least 10,000 events are needed to ensure that variance in the extracted count-rates is well below 10%. The results of this study are shown in Fig. 7. The lowest count rate that would be expected in an experiment is approximately 1 kHz. As a result, we determined that simulations should be run for 10 seconds of simulated time at each activity in order to ensure that 10,000 events are captured. As more events are recorded, the covariance of the extracted count rates decreases exponentially.

In order to contextualise the proposed for calibrating Monte Carlo detector models through evolutionary simulation to the existing methods, the ACCES-calibrated model is compared to the existing model described in Herald et al., (2021) through the ability to reproduce the real count-rate response of the ADAC Forte. This previous model was calibrated by using parameter values for the digitizer which were determined from the manufacturer’s characterisation and by manual calibration, taking a considerable amount of time and computational resources to achieve. The main advantage of using evolutionary simulation is the ability to achieve similar or, in this case, better results than manual calibration without spending the time and resources needed to run simulations, compare results, and update parameter values through iteration.

## Results and discussion

**Figure 8:**
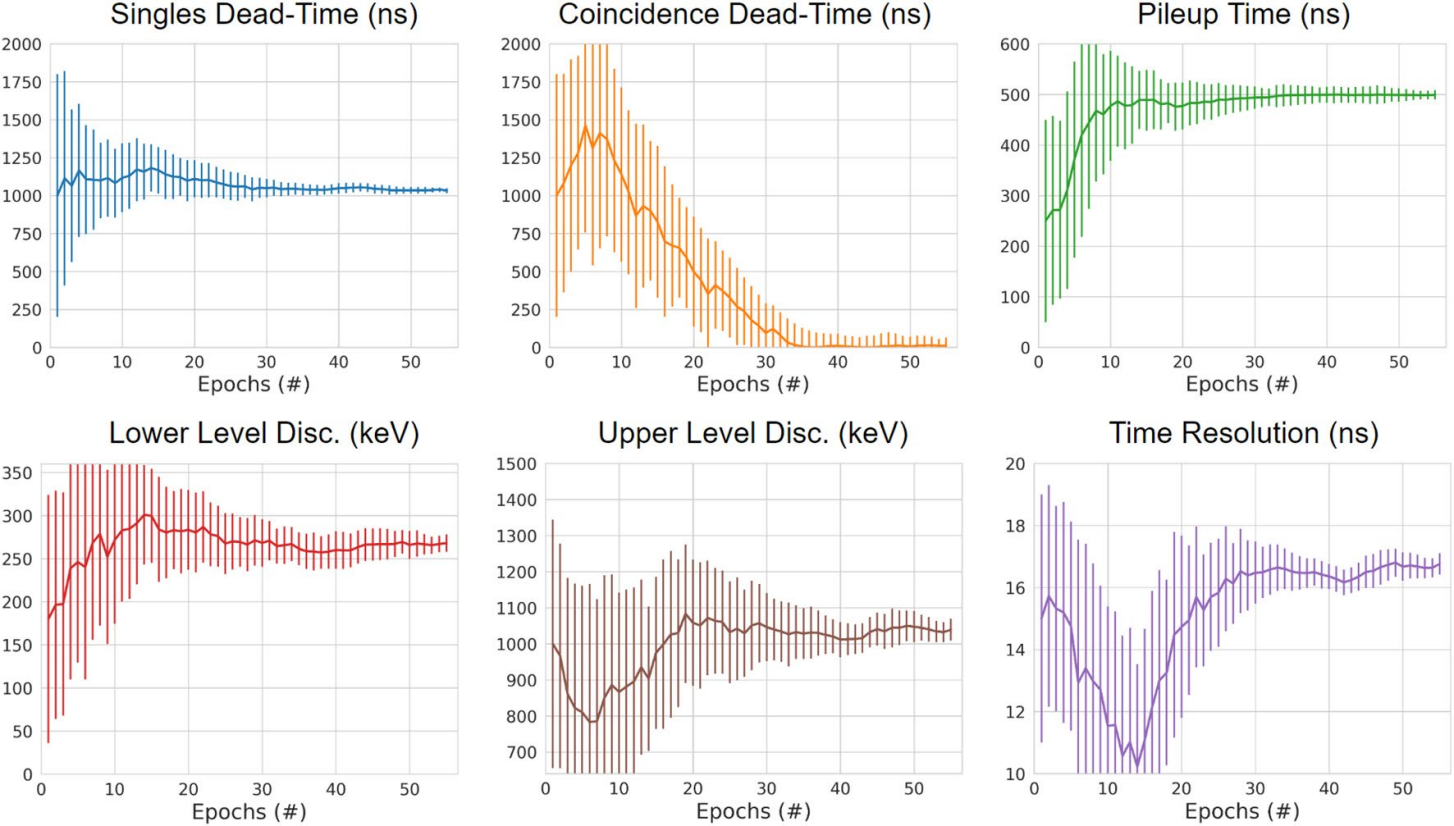
The mean parameter value guesses for each of the six free parameters with the standard deviation of the guesses are plotted as error bars. After 56 epochs all parameters are below 10% standard deviation and the optimisation is completed.

**Figure 9:**
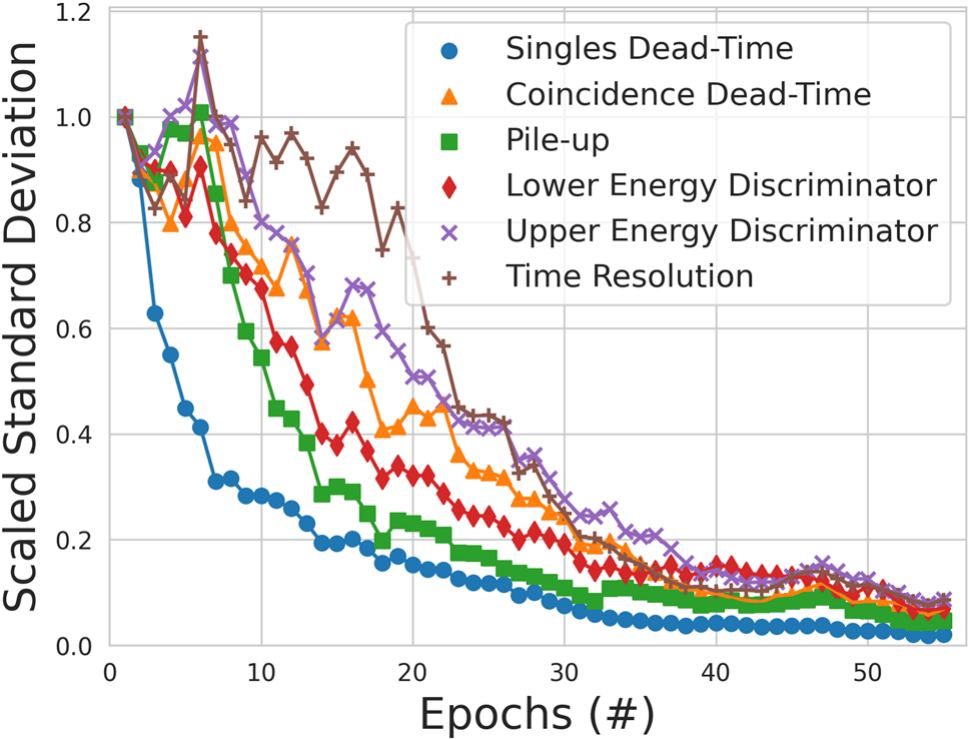
The standard deviation of the parameter value guesses tried by ACCES. A lower standard deviation represents lower uncertainty in discovering the optimal parameter values.

In total, the ACCES optimisation took 56 epochs, 8400 cost function evaluations, and approximately 4 days to complete. At the beginning of the ACCES optimisation, the guesses for the six free parameters are broad so as to explore the solution space. After this initial period, the guesses begin to converge to their optimal values as shown in Fig. 8 where the mean solution values and their standard deviations are plotted for the parameters over the optimisation. The scaled standard deviations are also shown in Fig. 9 to depict how the uncertainty in the optimisation decreases as the optimisation progresses.

Once the optimisation reached 10% uncertainty for each parameter, 0.1 scaled standard deviation, the parameters are considered calibrated. The final calibrated values are presented in Table 3. For all parameters except for the coincidence dead-time, the optimal solutions are well within their bounds, suggesting an optimal calibration that would not change with different bounds. However, the optimal coincidence dead-time is found to be approximately 0 ns. While this could be due to the bounds being ill-suited to the problem, in this case, we believe this demonstrates that coincidence dead-time is insignificant to the digitizer model. Further, support for this is that the model under-predicts the peak count rates. The opposite would be expected if coincidence dead-time was important.

**Table 3:** Calibrated digitizer parameter values.

Parameter	Calibrated value	Uncertainty
Singles dead-time (ns)	1070	± 16.7
Coincidence dead-time (ns)	10	± 54.7
Pile-up (ns)	498	± 9.31
Lower energy discriminator (keV)	284	± 10.1
Upper energy discriminator (keV)	1020	± 30.2
Time resolution (ns)	17	± 0.347

**Figure 10:**
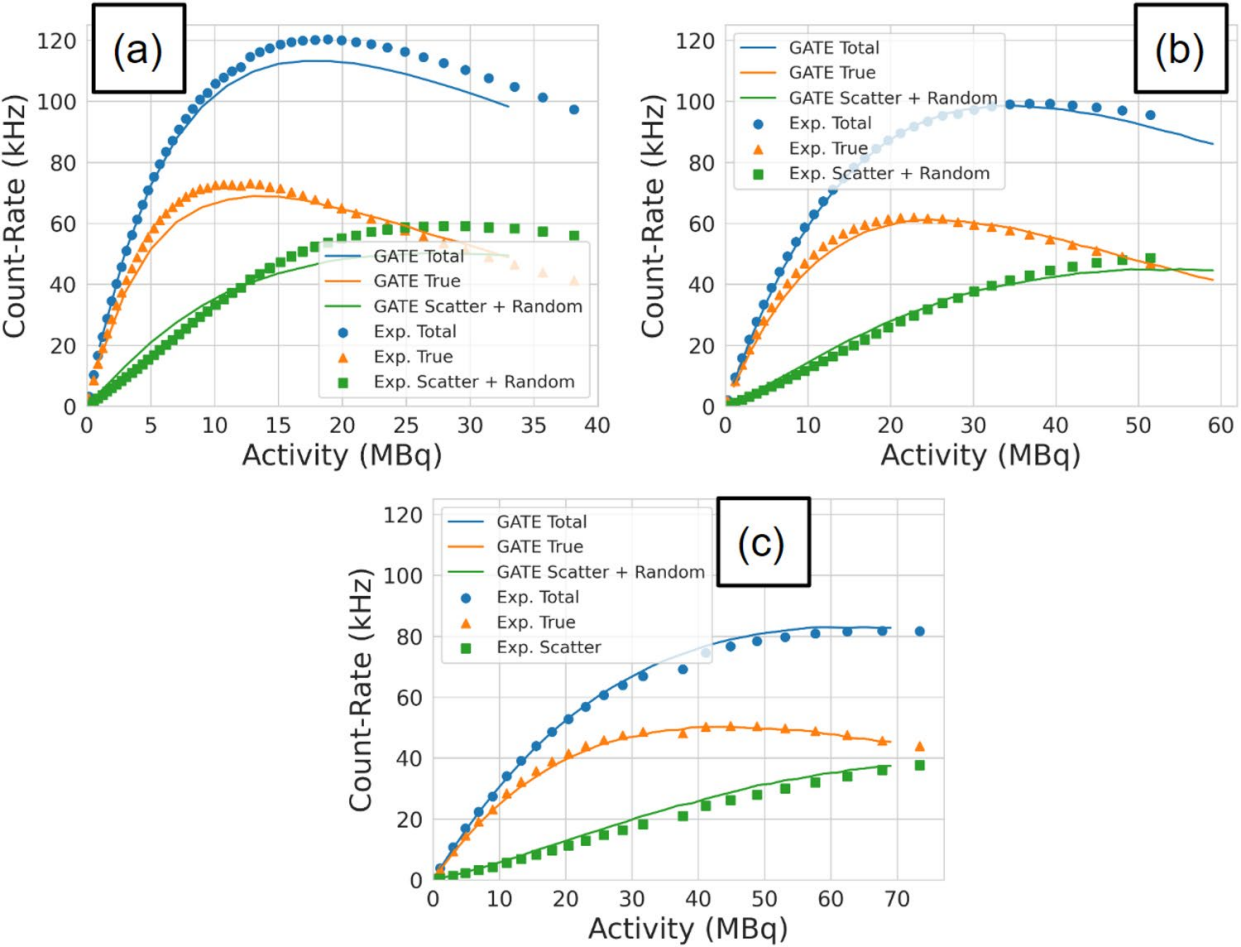
The optimised GATE model count rates are plotted against the experimental data for the (**a**) 250 mm experiment, (**b**) 525 mm experiment, (**c**) and the 800 mm experiment.

To assess the ability of the ACCES-calibrated digitizer model to replicate the experimental data, a new set of simulations is run for each head separation using the optimised values. After the simulations are finished, the results were plotted against the experimental data in Fig. 10. Visually, the count-rate response of the GATE model matches the general form of the real experiment. To quantify the accuracy, a mean absolute percent difference is calculated for each head separation and each type of count rate and presented in Table 4. Additionally, the results for the manually calibrated digitizer model are presented in Table 5.

**Table 4:** Mean absolute percent differences in the count rate of the ACCES-calibrated digitizer model.

ACCES-calibrated results			
Head separation	250 mm	525 mm	800 mm
Total count-rate	4.76%	1.71%	2.43%
True count-rate	4.55%	2.85%	2.25%
Scatter + random	13.33%	8.33%	11.77%
Average error	7.55%	4.30%	5.48%

**Table 5:** Mean absolute percent differences in the count rate of the manually calibrated digitizer model.

Manually calibrated results			
Head separation	250 mm	525 mm	800 mm
Total count-rate	11.50%	4.48%	6.25%
True count-rate	17.86%	15.68%	10.85%
Scatter + random	23.98%	26.10%	48.15%
Average error	17.78%	15.42%	21.75%

The average mean absolute percent differences for the 250 mm, 525 mm, and 800 mm are 7.55%, 4.30%, and 5.48%, respectively. The separation which was closest to the phantom experienced the highest error between the simulation and experiment. This could be caused by the closer separation amplifying differences between the phantom’s position in the simulation versus the experiment. In addition to this, the ACCES-calibrated model improves the match between simulation and experiment compared to a manually calibrated digitizer model which produced a mean absolute percent difference in the count rate response of 17.78%, 15.42%, and 21.75%. This represents ACCES producing a calibration which achieves a nearly three times better agreement with the experiments. This is a significant improvement and one accomplished without guiding the optimiser to these solutions. Overall, this calibration represents an agreement with the experiment that would be sufficient for the GATE model to be used as a predictive tool to generate data representative of real experiments.

**Figure 11:**
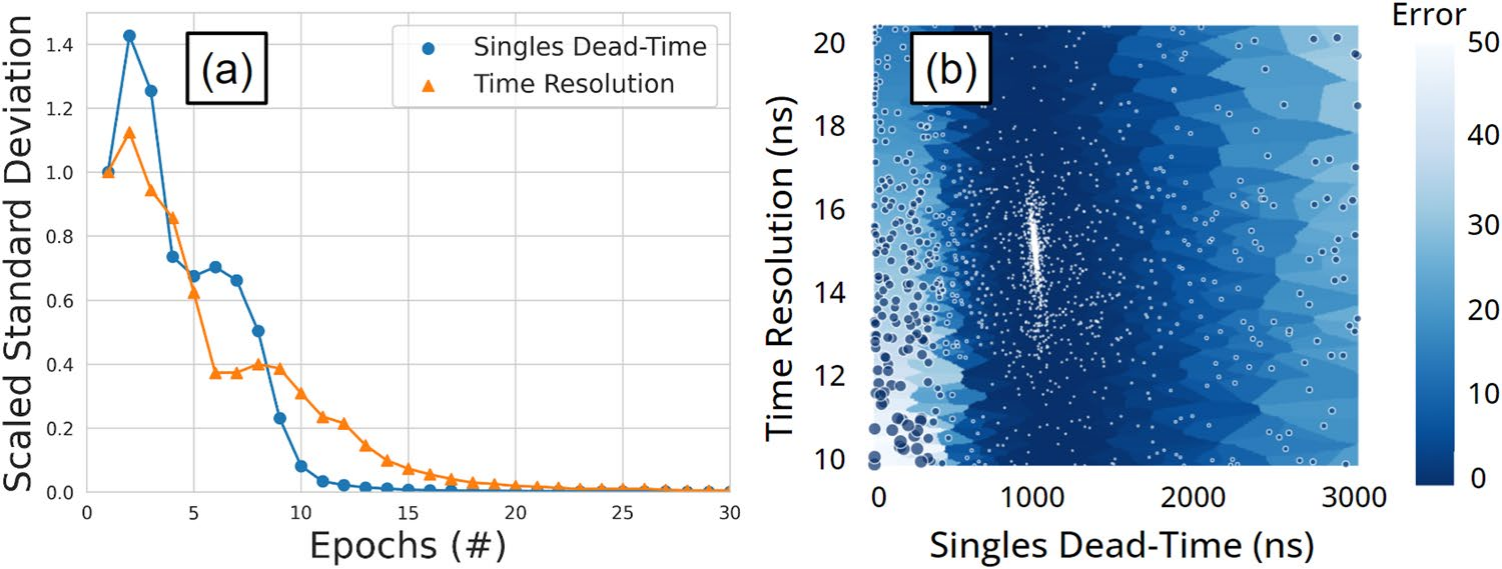
An example of an optimised GATE model with two free parameters. (**a**) The scaled standard deviation for each parameter over several epochs of simulations (**b**) a Voronoi diagram of the parameter combinations shows the solution converges to the optimal parameters. Each point is a candidate solution and the larger the point or lighter blue the Voronoi plot the higher the error in the cost-function evaluation.

To assist users in developing their own optimisations using ACCES, we have included an example within the GitHub repository found here. This example uses a simulated count-rate response of the ADAC Forte GATE model with prescribed parameter values in the digitizer as the ground truth response, then uses ACCES to calibrate two parameters, the singles dead-time and time resolution, to match the ground truth response. Two parameters were chosen because this is a more complex optimisation than a single parameter, yet easier to visualise than an optimisation with three or more parameters. The prescribed values for the singles dead-time and the time resolution are 1000 ns and 15 ns, respectively. The methodology in this simple example follows the same as that described in “Digitizer calibration” section. The results from this optimisation in Fig. 11 show the optimal parameter was determined to be 995.016 ns for the singles dead-time and 15.022 ns for the time resolution, which matches the prescribed parameters.

## Conclusions

In this work, we have demonstrated the calibration of a GATE digitizer model using an evolutionary algorithm. The model’s accuracy was quantified by a direct comparison of the ability of different parameter value combinations to replicate the count rate response of the detector across a diverse set of experiments. Importantly, the calibration was completed autonomously, needing only the number of simulations desired, the bounds of the search parameters, and the user-defined stopping criterion. This represents an advancement which brings simulations closer to reality. By employing the ACCES software available from our GitHub repository to perform this calibration, the need for users to perform a calibration through trial-and-error is eliminated. Even though this method needs a relatively long time and a large number of computational resources, the ability for ACCES to run on a high-performance computing system and periodically save the optimisation state makes this method useful and practical for users who have these resources at their disposal. While this workflow was demonstrated through the calibration of a specific detector model following the NEMA protocol, this same type of method can be applied to other models and also expanded to cover other types of measurements such as spatial resolution and sensitivity. Additionally, this method of optimisation can be improved in the future by including a strategy for calibrating the structure of the digitizer by including or excluding pulse-processing stages and by adjusting parameters which are categorical instead of quantitative, such as the type of dead-time model (paralyzable or non-paralyzable) or the policy for recording multi-coincidences.

## Data Availability

All data generated or analyzed during this study are included in this published article. Additionally, the ACCES software used for optimisation and the calibrated GATE model of the ADAC Forte have been made available through the University of Birmingham Positron Imaging Centre’s GitHub Repository: https://github.com/uob-positron-imaging-centre/ACCES-CoExSiST and https://github.com/uob-positron-imaging-centre/GATE_Models.
